# Novel strategy of remote magnetic navigation-guided ablation for ventricular arrhythmias from right ventricle outflow tract

**DOI:** 10.1038/s41598-020-75032-6

**Published:** 2020-10-20

**Authors:** Yun Xie, Ao Liu, Qi Jin, Ning Zhang, Kangni Jia, Changjian Lin, Tianyou Ling, Kang Chen, Wenqi Pan, Liqun Wu

**Affiliations:** Department of Cardiology, Shanghai Ruijin Hospital, Shanghai Jiao Tong University School of Medicine, No. 197, Ruijin Er Road, Shanghai, 200025 China

**Keywords:** Cardiology, Interventional cardiology

## Abstract

The optimized strategy to further increase the success rate of ablation for ventricular arrhythmias (VAs) from the right ventricular outflow tract (RVOT) is challenging. Recent studies have shown that the pulmonary sinus cusp (PSC) region may be the origin of certain RVOT VAs. We evaluated the efficacy of preferential ablation below the pulmonary valve (PV) and alternated radiofrequency delivery in the PSC using remote magnetic navigation (RMN). Sixty-five (65) consecutive patients experiencing VAs with RVOT-like appearance were included in this study. Mapping and ablation were preferentially performed below the PV. Ablation in the PSC would only be attempted when intensified ablation below the PV could not eliminate VAs. Finally, if ablation in the RVOT region failed, the aortic sinus cusp (ASC) would be mapped. Sixty-one (61) of 65 (93.8%) patients achieved procedural success. Except 7 cases of which the VAs were ablated in the ASC, the rest 54 VAs were thought to be originate from the RVOT region. Fifty (50) of 54 VAs were successfully ablated below the PV, and in the presence of a local special signal in the bipolar electrogram a more aggressive ablation was required. Subsequent ablation in the PSC with assistance of the RMN system achieved success in the remaining 4 patients. No complications occurred in this study. Our strategy of using RMN-guided ablation below the PV for VAs of RVOT origin was proved to be effective. PSC mapping and ablation using a magnetic catheter may provide the optimal strategy for treating these types of arrhythmias.

## Introduction

Premature ventricular complexes (PVCs) and ventricular tachycardia (VT) originating from the right ventricular outflow tract (RVOT) usually occur in patients without structural heart disease, can however be highly symptomatic and cause ventricular arrhythmia (VA)-induced cardiomyopathy, and are the most frequent forms of all idiopathic VAs^1^. Catheter ablation has been accepted as a curative treatment for patients with refractory RVOT VAs, as its procedural success rate is reported to be approximately 75–100% with a very low recurrence rate^2–4^. However, there have been reported cases in which complete eradication of RVOT VAs could not be obtained. While many authors have attempted to optimize the procedure through electrocardiographic or electrophysiological findings^5,6^, no consensus has been achieved. Some studies have shown that pulmonary sinus cusp (PSC) could also be the origin of VAs, including one conducted by Zhang et al.^7^ which suggested that over 90% of RVOT VAs could be ablated in the PSC. However, ablation in the PSC is generally considered to be difficult and risky as this region is relatively difficult to reach with a manual catheter. It is therefore still common practice that radiofrequency (RF) energy is firstly delivered below the pulmonary valve. It is thus critical to further optimize the ablation strategy for RVOT VAs, and to understand under which circumstances PSC ablation optimizes therapy.

Remote magnetic navigation (RMN) has been successfully used for the ablations of VAs^8^. We have previously reported our approach of RMN-guided catheter ablation for frequent PVCs originating from different locations, including the outflow tract^9,10^. With the soft-tipped catheter, RMN system was demonstrated in these studies to facilitate navigation to access difficult anatomic locations with superior stability and safety^11^. The purpose of our present study is to evaluate the efficacy of our strategy with preferential RMN-guided ablation below the pulmonary valve (PV) for all RVOT VAs. The application of RMN catheter manipulation with special morphology for ablation in PSC is also discussed.

## Methods

### Patient characteristics

In this study, 65 patients were consecutively enrolled at our institution between November 2017 and June 2019, with symptomatic and medically refractory VAs of likely RVOT-type origin (left bundle branch block morphology, inferior frontal axis, and precordial lead transition zone V3). None of the patients had received prior ablation therapy. Patients with structural heart disease including coronary artery disease, valvular heart disease or congenital heart disease were excluded from this study.

### Electrophysiological study

After withdrawal of antiarrhythmic drugs for five half-lives, electrophysiology studies were performed with patients in the fasting, conscious state, as previously described^9,10^. In all patients, a bi-polar catheter (St Jude Medical, Inc., St. Paul, MN) was placed at the apex of the right ventricle via the left femoral vein. Twelve-lead surface ECGs and intracardiac electrograms were recorded simultaneously by a digital multichannel mapping system, filtered at 30 to 400 Hz for bipolar electrograms and at 0.05 to 400 Hz for unipolar electrograms. If clinical PVCs failed to occur spontaneously, intravenous isoproterenol infusion (1–10ug/min) was administrated to induce VAs. A single bolus of 50–100 IU/kg body weight of heparin was administrated if mapping and ablation were necessary for the left ventricle outflow tract (LVOT) during the procedure. Additional heparin was administrated to maintain an activated clotting time between 200 and 250 s, as required.

### Mapping and ablation strategy

Patients underwent point-by-point electro-anatomical mapping using the CARTO 3D mapping system (Biosense Webster Inc., Irvine, CA) for RVOT region, following the standard protocol of our center^9,10^. An open-irrigated magnetic ablation catheter (NaviStar RMT ThermoCool, Biosense Webster Inc., Irvine, CA) was connected with the mapping system and the RMN Niobe ES and QUIK-CAS (Stereotaxis Inc., St. Louis, MO) to perform 3D LV electro-anatomic mapping and ablation. A Swartz sheath (SR0; St. Jude Medical Inc, St Paul, MN) was positioned between the inferior vena cava and tricuspid annulus via the right femoral vein, if necessary.

Activation mapping was initially performed below the pulmonary valve to identify the earliest activation site of the VA. We selected potential ablation sites where local activation was at least 20 ms pre-QRS with a QS wave in the unipolar electrogram. Pace mapping was also performed at these potential ablation sites. Best pace match was defined as a pacing QRS morphology matched in at least 11 of 12 leads. Bipolar potentials at the earliest activation site were analyzed afterwards. Sharp signals with multiple peaks were considered to be of importance, and the outcomes of ablation at these sites were further investigated in this study.

Radiofrequency energy was initially delivered below the pulmonary valve. Ablation was performed using power of 30 to 35 W and irrigation rate of 17 mL/min for 60 to 120 s. If complete eradication of VAs was not achieved, surrounding sites were further investigated by activation and pace mapping to find alternative ablation sites and subsequent ablations were applied to these sites. If VAs remained unchanged after multiple RF applications, PSC mapping and further ablation applications were then attempted. For patients with failed ablation in the RVOT and PSC region, mapping and ablation for the LVOT region with retrograde transaortic approach were attempted, which are not further discussed in this study. Acute success was defined as clinical VAs that were not induced with the administration of isoproterenol.

Clinical Time was recorded from the connection of the ablation catheter to sheath removal. Both Clinical Time and Ablation Time were recorded by the RMN system. Effective ablation sites were recorded by CARTO VISITAG (Biosense Webster, Inc., Irvine, CA). VISITAG points were automatically generated with a stability range of maximum 2.5 mm distance for a minimum of 8 s. Afterwards, total ablation area was calculated through these VISITAG points.

### Follow-up

Complications were recorded during the procedure and until hospital discharge. Continuous telemetry monitoring was performed for 24 h after the procedure for all patients. Patients were then scheduled for outpatient clinic visits 3 months post-procedure and every 6 months thereafter. A 24 h Holter monitor recording was performed within 6 months post-procedure. Recurrence of arrhythmia was defined as either symptomatic recurrence with documented VAs or asymptomatic frequent PVCs numbering over 5,000 per day^12^.

### Statistical analysis

Continuous variables were expressed as mean ± SD, and categorical variables as a percentage. An unpaired Student's *t* test was used to compare the continuous variables between two groups. For the comparison among 3 groups, ANOVA test was performed, with Tamhane’s T2 test for further post hoc analysis. A value of *p* < 0.05 was considered statistically significant. SPSS 26.0 software was used for all of the statistical analysis.

### Ethics approval

The study was approved by the ethics board of Ruijin Hospital, Shanghai Jiao Tong University School of Medicine, and complies with the Declaration of Helsinki. All the patients included in this study signed an informed consent before the procedure.

## Results

### Patient characteristics

Baseline characteristics of the enrolled patients are listed in Table 1. The average age of the patients was 47.8 ± 14.5 years old, while 29.2% of them were male. All patients had symptomatic VAs, with an average VA burden of 20,840.3 ± 10,017.6 on 24-h Holter monitoring, and had a history of failed medication therapy.

**Table 1:** Patient characteristics.

	Total
	N = 65
Age (year)	47.8 ± 14.5
Male	19 (29.2)
LVEDd (mm)	48.5 ± 4.2
LVEF (%)	65.1 ± 4.9
Hypertension	19 (29.2)
Type 2 diabetes	5 (7.7)
VT	15 (23.1)
VA burden on 24 h Holter	20,840.3 ± 10,017.6
Number of prescribed AADs	1.9 ± 0.8
Values are n(%) or mean ± SD	

*LVEDd* left ventricular end-diastolic diameter, *LVEF* left ventricular ejection fraction, *VA* ventricular arrhymia, *AAD* antiarrhythmic drug.

**Figure 1:**
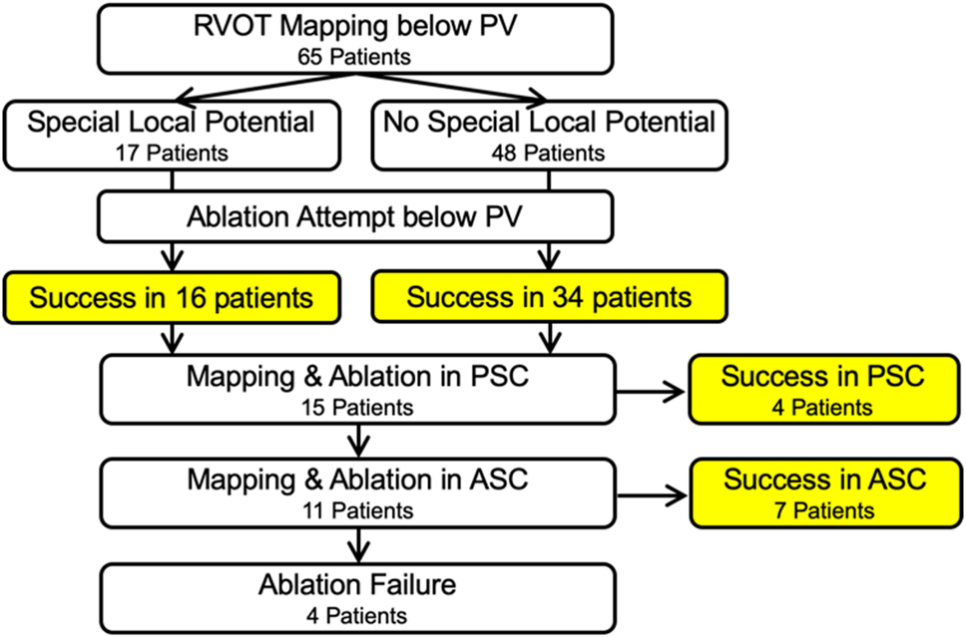
Flow chart of mapping and ablation results.

The flow chart of mapping and ablation results is shown in Fig. 1. Acute ablation success was achieved in 61 of 65 (93.8%) patients. Among them, 54 procedures were successfully ablated in the RVOT region, including the sites below PV and in PSC. For 7 patients, acute success was achieved after ablation of VAs in the aortic sinus cusp (ASC) region, while only 4 patients resulted in ablation failure. The electrophysiologic characteristics and ablation of the 54 RVOT VA cases in which ablation succeeded were further investigated in this article.

### Electrophysiology and ablation findings below PV

For all the patients enrolled in the study, RVOT mapping was performed first. The site with the earliest activation site was initially assessed as the most probable VA origin. For the 50 patients in which acute success was achieved below the PV, 42 (84%) patients were with VAs from the RVOT septal area, with the remaining VAs originating from the RVOT free wall. The average activation time prior to QRS was 33.9 ± 7.9 ms. Pace mapping was generally good at these sites, and a unipolar QS configuration was always presented.

**Figure 2:**
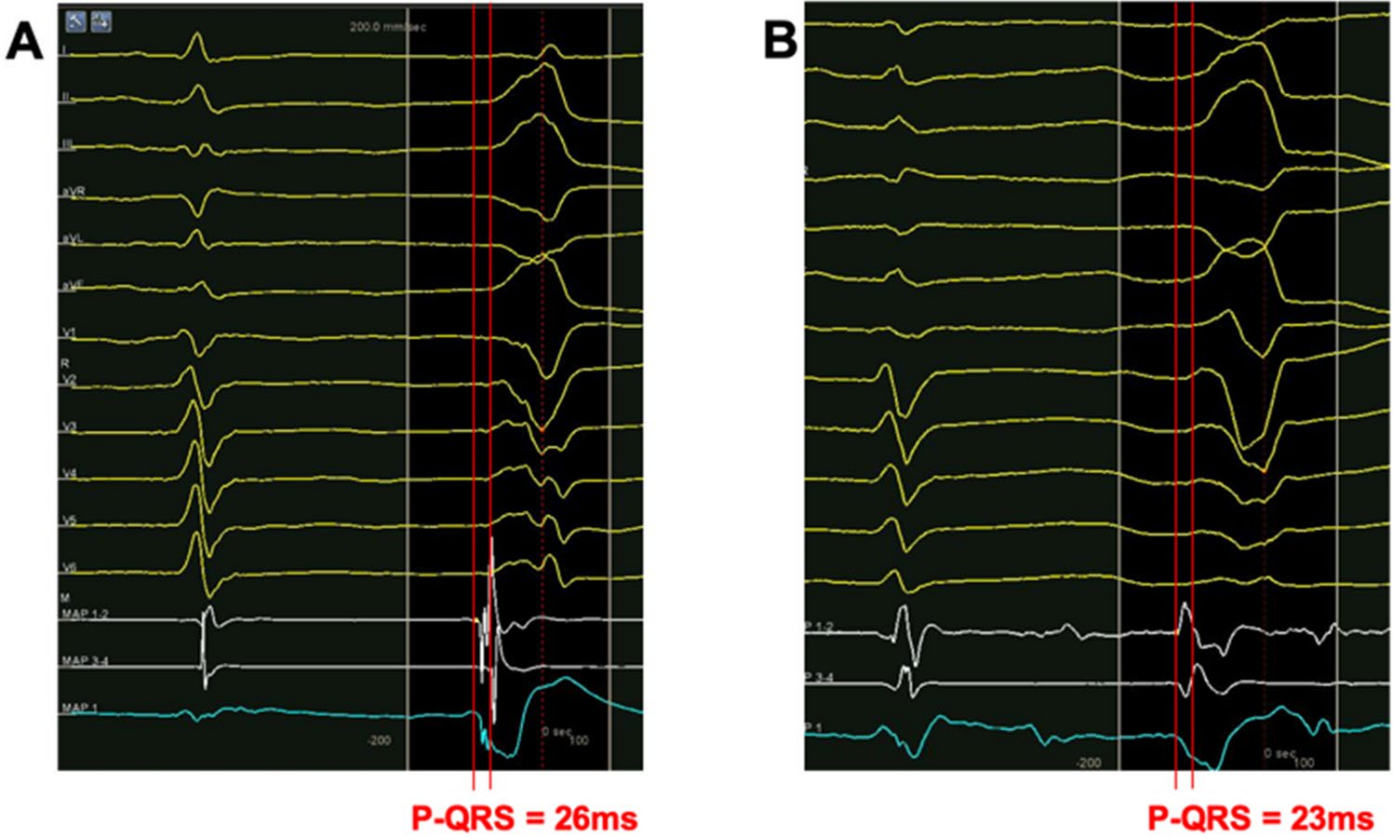
Bipolar and unipolar electrogram at the site with earliest activation time below PV. (**A**) The point with special potential, (**B**) the point without special potential. In both figures, bipolar electrograms of distal pair were presented as the upper white line.

**Figure 3:**
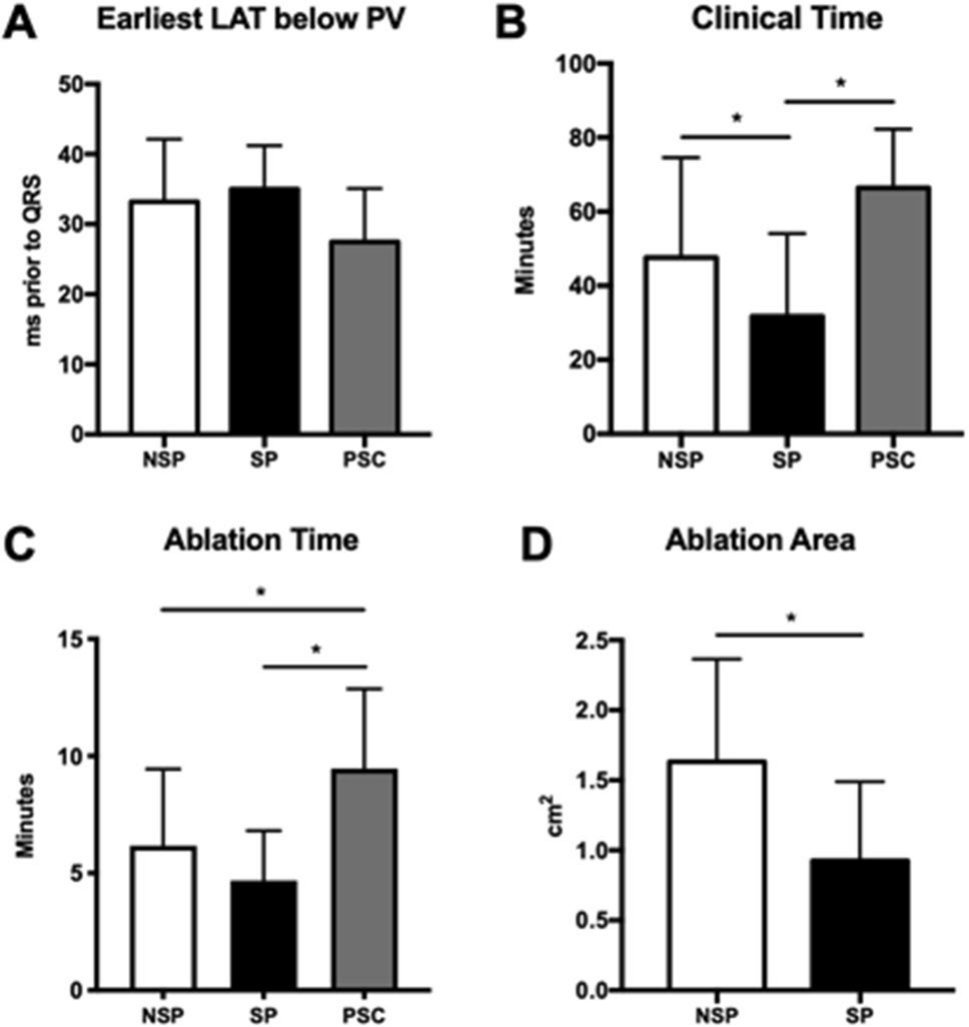
The influence of the special potential appearance to multiple mapping and ablation parameters including: (**A**) Earliest LAT at the site below the PV; (**B**) clinical time of the whole procedure; (**C**) total ablation time; (**D**) ablation area. NSP = Group of VAs with no special potential; NSP = Group of VAs with special potential; VAs of both NSP and SP group were successfully ablated below PV. PSC = Group of VAs that were finally eliminated in PSC. For all figures, *p < 0.05.

However, despite those similarities, the local potential in bipolar electrogram varied among cases, which may predict the outcome of RF energy delivery in these sites. We investigated all of the local potentials at the earliest activation site below the PV. In 17 of 65 (26%) patients, the local bipolar potentials were sharp, with multiple peaks (Fig. 2A). Sixteen (16) out of 17 patients achieved acute success below the PV, and were defined as having special potential (SP group). In other cases, no special potential was found below the PV. Those 34 patients with no special potential but whose VAs were also ablated below the PV were classified as ones with no special potential (NSP group, Fig. 2B). The local activation time (LAT) was 35.0 ± 6.2 ms prior to QRS in SP group, with a similar time of 33.2 ± 8.9 ms (Fig. 3A) in the NSP group.

**Figure 4:**
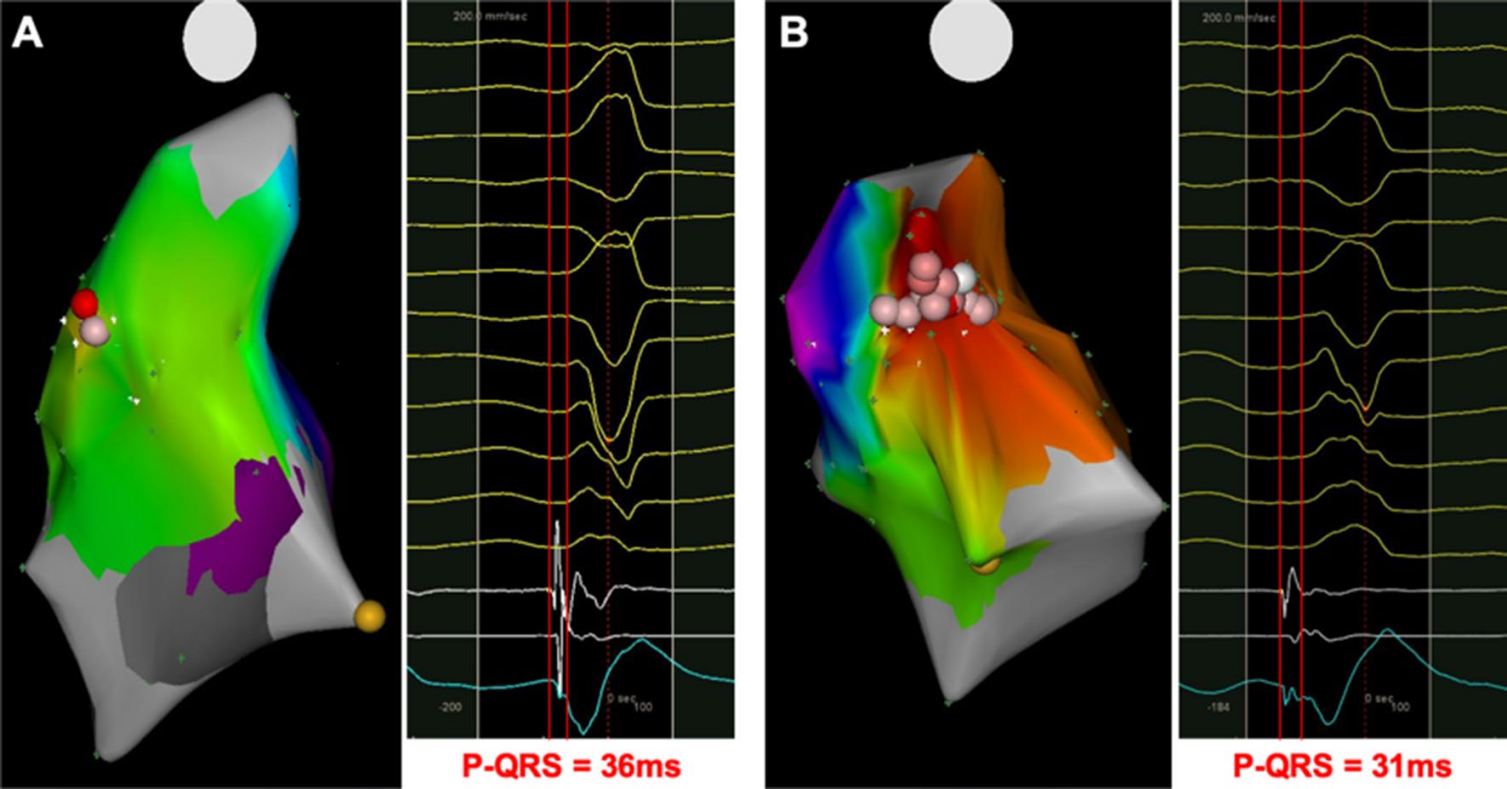
Examples of successful ablation below the PV in VAs with (**A**) or without (**B**) special potential. In each figure, the left panel shows the CARTO activation map of a PVC in the posterior–anterior view, while the local electrogram at the initial ablation site is shown in the right panel.

For safety precautions and relatively simplistic approach, RF energy was always initially delivered below the PV, disregarding the local bipolar electrogram morphology. Multiple ablations would be attempted in the cases that VAs were not completely eliminated. Examples of ablation procedure for both SP and NSP group are shown in Fig. 4. By reviewing these cases, we found that among all the 50 cases which ablation below the PV succeeded, the clinical time of the SP group was significantly shorter than that of the NSP group (31.8 ± 22.3 min in SP vs. 47.6 ± 27.0 min in NSP, *p* < 0.05, Fig. 3B). Similarly, total ablation time was also lower in the SP group, though no statistical difference was observed between the two groups (4.7 ± 2.1 min in SP vs. 6.2 ± 3.3 min in NSP, *p* = 0.11, Fig. 3C). Nonetheless, in the SP group, the total ablation area for the eradication of VAs was significantly decreased (0.92 ± 0.56 cm^2^ in SP vs. 1.63 ± 0.73 cm^2^ in NSP, *p* < 0.05, Fig. 3D). All of these data indicated that the sites with the special local bipolar potential could be nearer to the origin of VAs and were thus prone to be eliminated by RF ablation. VAs with no special bipolar potential likely originated from other nearby structures, but additional ablation sets in surrounding areas could still lead to acute success in part of the cases.

### RF ablation in PSC with RMN system

**Figure 5:**
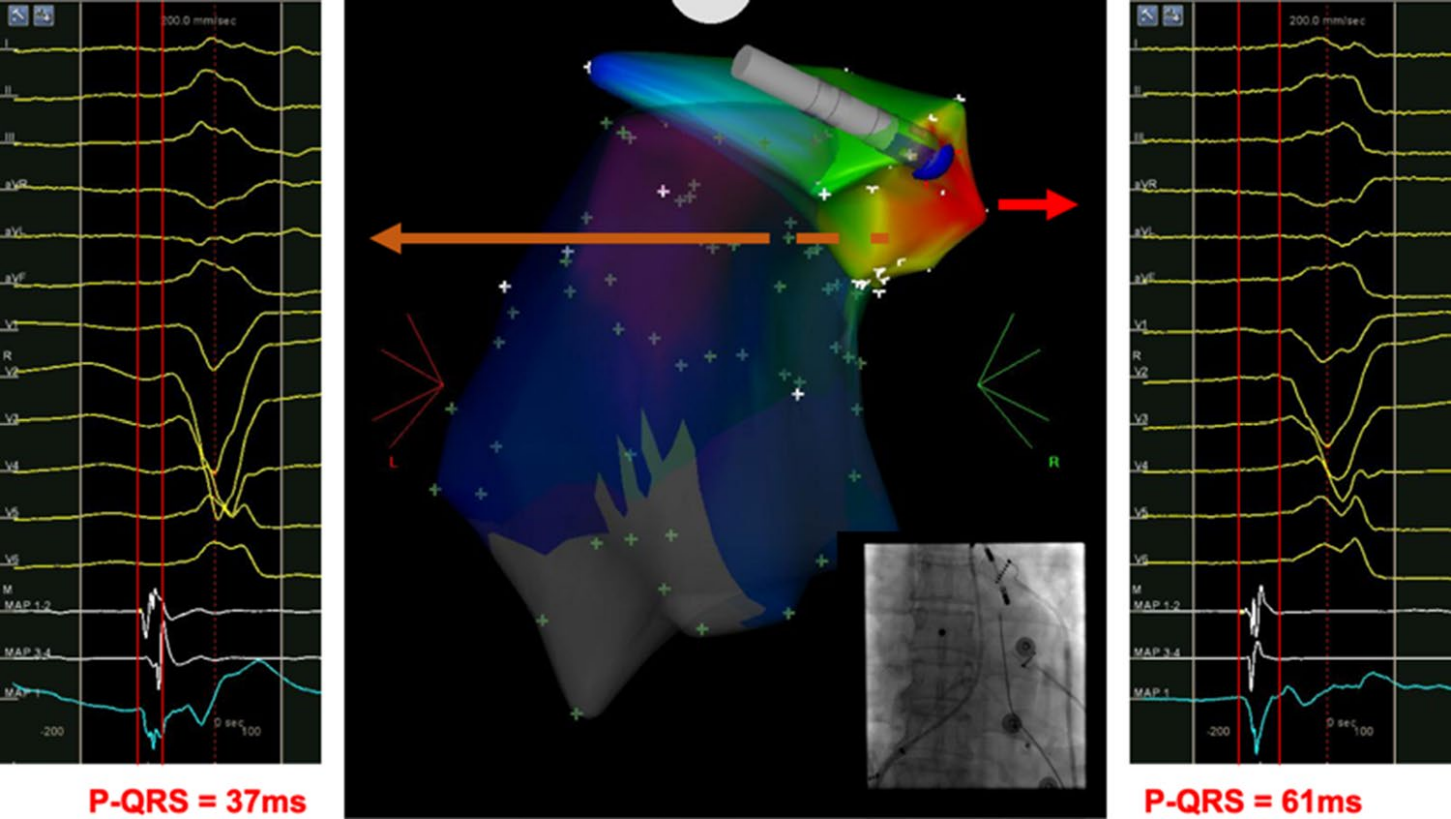
An example of successful ablation in the PSC. In the left panel, the local electrogram of the site with earliest activation time below the PV is presented. In the middle: the CARTO activation map of a PVC in the posterior–anterior view is shown, while fluoroscopic view in the anterior–posterior view is also presented in the bottom right corner. The blue dot points to the site where ablation led to the elimination of PVCs. The local electrogram of the site with earliest activation time during PSC mapping is presented in the right panel.

In the remaining 15 patients where ablation below the PV was not successful, PSC mapping and ablation was attempted. To facilitate the procedure, the ablation catheter, guided by the RMN system, firstly formed a ‘reversed U curve’ in the pulmonary artery (PA) and was then directed downward to reach the PSC region. In four patients, the LAT was found to be earlier in the PSC region than that below the PV, and their VAs were subsequently ablated successfully at that site, as the example illustrated in Fig. 5. In these 4 cases, their LATs prior to QRS at sites below the PV were slightly later than that of the cases with successful ablation. To no surprise, the clinical time and burns time were significantly longer (Fig. 3A–C). This suggests the efficacy of the RMN system for mapping and ablation of VAs originated from the PSC. However, in the other 11 cases, 7 required ablation in the ASC region, indicating that VAs originated from the PSC could only explain why ablation below the PV resulted in a failure in part of the cases.

### Follow-up

The average follow-up was 14.4 ± 5.6 months after the procedure. No procedure-related complications such as cardiac tamponade occurred during or after ablation in any of the 65 patients. In the 61 patients who had a successful ablation, only 2 (3.3%) reported recurrence of VAs, both having received ASC ablation in the initial procedures. Worsening of tricuspid or pulmonic regurgitation and new onset of coronary artery stenosis were not found in any of the 65 patients.

## Discussion

### Main findings

Catheter ablation successfully eliminated 61 of 65 VAs with suspected RVOT origin, with only 2 cases of long-term recurrence. Among them, 54 of 61 PVCs or VTs were ablated in the RVOT region, with a majority of them being eliminated below the PV. For the rest of the patients, PSC mapping and ablation with guidance of the RMN system proved to be a practical subsequent approach. VAs originating in the PSC region comprised a small portion (4 of 15 cases) of cases with failed ablation below the PV. No critical complications occurred during or after the procedure. These data suggested our ablation strategy for idiopathic RVOT VAs using the RMN system are extraordinarily effective and safe.

### Mapping and ablation strategy for RVOT VAs

Catheter ablation for RVOT VAs have been reported to have a success rate of nearly 90%. A better ablation outcome depends on the amelioration of mapping strategies to determine VA origin with improved precision. Typically, mapping procedures are guided by unipolar and bipolar electrograms. Today, most RF catheter ablation procedures for focal arrhythmias are guided by LAT defined by the initial peak of the bipolar electrogram, then confirmed by the ‘QS’ form of the unipolar electrogram and by pace mapping. However, unipolar electrograms provide information about a large area and contain certain far-field signals, resulting in a broad QS configuration. Pace mapping in the RVOT region has also shown to have inferior resolution when compared to activation mapping^13^. Similarly, in our study, pace mapping and unipolar electrogram aligned at the site with the earliest LAT, concluding that other than LAT, little information can be provided through current mapping techiniques.

In the attempt to optimize the procedure, multiple studies have focused on the bipolar electrogram at the earliest activation site in the RVOT region. van Huls van Taxis et al.^6^ discovered that, at the site with earliest LAT, the presence of reversed polarity, which was evaluated from the distal and mid electrode pairs of mapping catheters, was a good predictor of ablation success. Recently, Parreira et al.^14^ reported that the presence of diastolic potentials at the ablation site, which may result from delayed afterdepolarization, was associated with success. A seperate study conducted by Lee et al.^15^ suggested late fractionated potentials in unipolar electrogram as a novel predictor. However, due to either the significant increase in the complexity of the procedure, or the relatively low presence of the indicated signals, these strategies were not feasible in typical ablation procedures. In this study, we adopted a simpler classification to focus on the steep signals with multi-peaks, which were conventionally considered ‘good’, as a special potential, which proved to be associated with a narrower ablation area and better ablation outcome. However, special potentials only presented in approximately one thirds of the cases. A more precise definition of this potential, along with the strategy for cases with no special potential, requires further investigation to provide greater clarity.

PSC mapping and ablation has recently been regarded as a possible origin of RVOT VAs^7,16^. Its theoretic support comes from the discovery that the right ventricle musculature could extend into the pulmonary artery between the cusps and into the valve tissue itself in a sleeve-like manner^17^. As mentioned above, one study suggested that ablation in PSC alone could eliminate 90% of RVOT VAs. To prove the efficacy of this strategy, larger scale trials should be conducted. In the interim, safety issues are a concern, as drastic movement of the relatively stiff manual catheter in PA and RF delivery in PSC are generally considered more risky, especially for the novice operator. In our opinion, for RVOT VAs, mapping and ablation below PV should still be the first choice, considering current evidence in this field.

As the identification of RVOT VAs have a relative specificity and sensitivity according to the characteristic of standard 12-lead ECG, VAs with failed ablation in the RVOT region or PSC may originate from neighboring structures. Since the septum of the RVOT is located adjacent to the anterior left coronary cusp and right coronary cusp, mapping of the ASC region should be considered^18^. In this study, 7 out of 11 patients with failed ablation in the RVOT region finally achieved preferred outcome in the ASC. However, RF delivery for VAs arising from the ASC should be done with extreme caution and coronary angiography is required to minimize the potential risk of coronary artery injury.

Taking both efficacy and safety into account, our initial approach is to attempt ablation below the PV in patients without special local potential. We have shown that most RVOT VAs can still be eradicated in this group with intensified ablation, which was likely due to multiple sleeve-like extensions connected to the VA origin near PSC. Only 4 cases required PSC ablation. Ablation in the ASC was successful in the other 7 patients, suggesting that some of the VAs without special potential were of non-RVOT origin. For all of these patients, our strategy was proved to be effective and practical. The question is whether mapping ASC or PSC before determination of ablation site could achieve similar outcome and further reduce ablation area in these cases. Prospective trials of a larger scale should be conducted to provide solid evidence for this question.

### Ablation below PV and in PSC with guidance of RMN system

The RMN system has been proven to be able to provide more precise catheter movement with more stability in the treatment of most arrhythmias^19^. RMN-guided ablation of RVOT VAs was demonstrated to achieve comparable success rate with less overall fluoroscopic time, compared to the conventional method^20,21^. In addition, lesser pro-arrhythmia effects of the magnetic catheter decreased the incidence of mechanical PVCs and increased the mapping precision, thus, decreasing the number of ineffective ablation applications.

While the efficacy of the RMN system has been widely published, previous studies have never investigated the local electrogram recorded by the RMN-guided catheter. With its superior reachability, the catheter is more able to get closer to the junction of the muscular infundibulum and the pulmonic valve, a probable origin of distal RVOT VAs. Whether the special potential we have seen in the study can be more frequently found in RMN-guided mapping can be an additional interesting topic of research.

Safety is another advantage for RMN-guided ablation. It is especially important for free wall-origin RVOT VAs, as ablating there carries a higher risk for cardiac tamponade than in the septal region. In our previous study^9^, manual control navigation was shown to be responsible for 3 cases of cardiac tamponade. All 3 cases occurred during ablation of free wall-origin RVOT VAs. In contrast, until now, no such complication has occured in our center when ablating RVOT VAs with assistance of the RMN system.

To our knowledge, there is no published report regarding PSC ablation with the RMN system. While not generally used in our strategy, we have proved in this study that RMN-guided mapping and ablation in the PSC can be achieved by manipulating the catheter with the ‘reversed U-curve’ method. In our opinion, the soft tip of the magnetic catheter could simplify its maneuver in the PA, with superior safety, thus potentially facilitating successful ablation procedure in the PSC.

### Study limitations

There are several limitations in the current study. First, intracardiac echocardiography was not used to further identify catheter location. Second, the classification of the special potential in this study in some circumstances is ambiguous, which requires further quantification through the assessment of additional cases. Nevertheless, the feasibility of our current strategy and the validity of the special potential on bipolar electrogram should be further evaluated in randomized controlled trials.

## Conclusions

Our strategy of RMN-guided catheter ablation for RVOT VAs proved to be of great efficacy and safety. Ablation below the PV could be effective for most VAs of RVOT origin. For those failed with intensified ablation, PSC mapping and ablation could be a supplementary methodology, which could also be facilitated by magnetic catheter directed by the RMN system.

## References

[1] John RM, Stevenson WG (2016). Outflow tract premature ventricular contractions and ventricular tachycardia: the typical and the challenging. Card. Electrophysiol. Clin..

[2] Cronin EM, Bogun FM, Maury P, Peichl P, Chen M, Namboodiri N (2019). HRS/EHRA/APHRS/LAHRS expert consensus statement on catheter ablation of ventricular arrhythmias. Europace.

[3] Shivkumar K (2019). Catheter ablation of ventricular arrhythmias. N. Engl. J. Med..

[4] Ventura R, Steven D, Klemm HU, Lutomsky B, Mullerleile K, Rostock T (2007). Decennial follow-up in patients with recurrent tachycardia originating from the right ventricular outflow tract: electrophysiologic characteristics and response to treatment. Eur. Heart J..

[5] Baser K, Bas HD, Belardi D, Yokokawa M, Good E, Latchamsetty R (2014). Predictors of outcome after catheter ablation of premature ventricular complexes. J. Cardiovasc. Electrophysiol..

[6] van Huls van Taxis, C. F., Wijnmaalen, A. P., den Uijl, D. W., Gawrysiak, M., Putter, H. & Schalij, M. J. et al. Reversed polarity of bipolar electrograms for predicting a successful ablation site in focal idiopathic right ventricular outflow tract arrhythmias. *Heart Rhythm*. 2011;8(5):665–71. 10.1016/j.hrthm.2010.12.049. 10.1016/j.hrthm.2010.12.049 21215326

[7] Zhang J, Tang C, Zhang Y, Su X (2018). Pulmonary sinus cusp mapping and ablation: a new concept and approach for idiopathic right ventricular outflow tract arrhythmias. Heart Rhythm..

[8] Aagaard P, Natale A, Briceno D, Nakagawa H, Mohanty S, Gianni C (2016). Remote magnetic navigation: a focus on catheter ablation of ventricular arrhythmias. J. Cardiovasc. Electrophysiol..

[9] Qiu X, Zhang N, Luo Q, Liu A, Ji Y, Ye J (2018). Remote magnetic navigation facilitates the ablations of frequent ventricular premature complexes originating from the outflow tract and the valve annulus as compared to manual control navigation. Int. J. Cardiol..

[10] Xie Y, Jin Q, Zhang N, Liu A, Xing C, Jia K (2019). Strategy of catheter ablation for para-Hisian premature ventricular contractions with the assistance of remote magnetic navigation. J. Cardiovasc. Electrophysiol..

[11] Bradfield J, Tung R, Mandapati R, Boyle NG, Shivkumar K (2012). Catheter ablation utilizing remote magnetic navigation: a review of applications and outcomes. Pacing Clin. Electrophysiol..

[12] Mountantonakis SE, Frankel DS, Gerstenfeld EP, Dixit S, Lin D, Hutchinson MD (2011). Reversal of outflow tract ventricular premature depolarization-induced cardiomyopathy with ablation: effect of residual arrhythmia burden and preexisting cardiomyopathy on outcome. Heart Rhythm..

[13] Bogun F, Taj M, Ting M, Kim HM, Reich S, Good E (2008). Spatial resolution of pace mapping of idiopathic ventricular tachycardia/ectopy originating in the right ventricular outflow tract. Heart Rhythm..

[14] Parreira L, Marinheiro R, Carmo P, Amador P, Mesquita D, Farinha J (2019). Isolated diastolic potentials as predictors of success in ablation of right ventricular outflow tract idiopathic premature ventricular contractions. PLoS ONE.

[15] Lee WC, Wu PJ, Fang HY, Chen HC, Chen YL, Tsai TH (2019). Late fractionated potentials in catheter ablation for right ventricular outflow tract ventricular arrhythmias. Pacing Clin. Electrophysiol..

[16] Liao Z, Zhan X, Wu S, Xue Y, Fang X, Liao H (2015). Idiopathic ventricular arrhythmias originating from the pulmonary sinus cusp: prevalence, electrocardiographic/electrophysiological characteristics, and catheter ablation. J. Am. Coll. Cardiol..

[17] Gami AS, Noheria A, Lachman N, Edwards WD, Friedman PA, Talreja D (2011). Anatomical correlates relevant to ablation above the semilunar valves for the cardiac electrophysiologist: a study of 603 hearts. J. Interv. Card. Electrophysiol..

[18] Yamada T (2018). Which ventricle should be mapped first in catheter ablation of ventricular arrhythmias originating from the ventricular outflow tract?. J. Cardiovasc. Electrophysiol..

[19] Aagaard P, Natale A, Di Biase L (2015). Robotic navigation for catheter ablation: benefits and challenges. Expert Rev. Med. Devices.

[20] Parreira L, Cavaco D, Reis-Santos K, Carmo P, Cabrita D, Scanavacca M (2013). Remote magnetic navigation for mapping and ablation of right and left ventricular outflow tract arrhythmias. Rev. Port Cardiol..

[21] Zhang F, Yang B, Chen H, Ju W, Kojodjojo P, Cao K (2013). Magnetic versus manual catheter navigation for mapping and ablation of right ventricular outflow tract ventricular arrhythmias: a randomized controlled study. Heart Rhythm..

